# Effects of yam dioscorin interventions on improvements of the metabolic syndrome in high-fat diet-induced obese rats

**DOI:** 10.1186/s40529-015-0084-8

**Published:** 2015-02-25

**Authors:** Shen-Liang Shih, Yin-Shiou Lin, Shyr-Yi Lin, Wen-Chi Hou

**Affiliations:** 1 Yuan’s General Hospital, Department of Breast Surgery and Cancer Center, Kaohsiung, Taiwan; 2 Graduate Institute of Pharmacognosy, Taipei Medical University, Taipei, Taiwan; 3 Department of Primary Care Medicine, Taipei Medical University Hospital, Taipei, Taiwan; 4 Department of General Medicine, School of Medicine, Taipei Medical University, Taipei, Taiwan; 5 Traditional Herbal Medicine Research Center, Taipei Medical University Hospital, Taipei, Taiwan; 6 Program for the Clinical Drug Discovery from Botanical Herbs, Taipei Medical University, Taipei, Taiwan

**Keywords:** Dioscorin, Dipeptidyl peptidase IV (DPP IV), Metabolic syndrome (MS), Peptic hydrolysates, Systolic blood pressure, Yam

## Abstract

**Background:**

The metabolic syndrome (MS) is termed a cluster of multiple metabolic risk criteria which is positively correlated with cardiovascular disease and type 2 diabetes mellitus (DM). Yam dioscorins have been reported to exhibit biological activities, however, little is known their preventive effects on the MS. Therefore, a high-fat (HF) diet was used to induce Wistar rat obesity and then yam dioscorin (50 mg/kg, dio50) was intervened daily concurrent HF diet (HF diet + dio50) for five weeks to check the changes of weights of body and tissues, blood pressures, and impaired glucose tolerances. The in vitro peptic hydrolysates of dioscorin with molecular mass between 3 kDa and 10 kDa and less than 3 kDa were used to determine dipeptidyl peptidase IV (DPP IV) inhibitory activities which DPP IV inhibitor has been reported to prevent and treat type 2 DM.

**Results:**

There were no significant difference in body weights, feed intakes, feed conversion, and weights of adipose tissues of obese rats in groups of HF and (HF diet + dio50). However, the systolic blood pressures in obese rats of 2-, 3- and 4-week dioscorin interventions were showed significantly lower (*P* < 0.05) compared to the HF group. The dioscorin intervention (HF+ dio50) was showed significantly different (*P* < 0.05) and improved the impaired glucose tolerances compared to HF group in obese rats by the oral glucose tolerance tests. It was also found that the fraction with different molecular mass of dioscorin peptic hydrolysates (5 mg/ml) showed inhibitory activities against DPP IV using sitagliptin phosphate as positive controls.

**Conclusions:**

Yam dioscorins exhibit improved MS activities in obese rats which the related mechanisms may need further investigations.

**Electronic supplementary material:**

The online version of this article (doi:10.1186/s40529-015-0084-8) contains supplementary material, which is available to authorized users.

## Background

The metabolic syndrome (MS) is termed a cluster of five multiple metabolic risk criteria, including (1) abdominal obesity (central obesity), (2) hypertriglyceridemia [fasting triglycerides (TGs) ≥ 150 mg/dl], (3) low serum high-density lipoprotein cholesterol (HDL ≤ 40 mg/dl), (4) elevated blood pressure (systolic ≥ 130 mmHg or diastolic ≥ 85 mmHg), and (5) high fasting blood glucose (≥110 mg/dl), and an increasing obesity and sedentary lifestyles relate positively the rising MS worldwide prevalence (Day [2007]; Alberti et al. [2009]). A general diagnosis with three out of above-mentioned five criteria can recognize as the MS (Day [2007]). Insulin resistance and impaired glucose tolerance are also two important metabolic risk criteria which may involve in factors for the MS general diagnosis (Day [2007]). The health risks of MS associated with obesity vary among individuals, but consistently include type 2 diabetes mellitus (type 2 DM), hypertension, coronary heart disease and cancer (Zimmet [1982]; Prentice [2006]). The abdominal obesity may play the central role in MS which is the situation of exceeding visceral fat deposited in peritoneal cavity, and will initiate inflammation and dyslipidemia, increase the blood pressure and decrease insulin sensitivity accompanied with abnormal blood glucose. Obesity is blamed as a major contributing factor in over 0.3 million deaths per year in the America and related economic costs over US 100 billion per year (Daniels [2006]; Rodgers et al. [2012]). In fact, obesity associated with diabetes are considered not only a clinical problem but also a public health issue in many countries, and 80% of overweight people are also diagnosed with type 2 DM which are referred as “the twin epidemics” (Smyth and Heron [2005]).

Several animal models are established and suitable for one or more metabolic risk criteria in MS studies (Panchal and Brown [2011]), such as genetic rodent models, including *db/db* mice, *ob*/*ob* mice, Zucker diabetic fatty rats, and Otsuka Long-Evans Tokushima fatty (OLETF) rats etc., however, such genetic mutations in established rodent animals are few reported in humans; on the other hand, the diet-induced rodent models may mimic closely to MS symptoms in humans, including fructose-induced, sucrose-induced, and high-fat (HF) diet induced rodent animals (Panchal and Brown [2011]). The HF diets are reported to induce overweight, obesity, dyslipidemia, insulin resistance, and high blood pressures in rodent animals (Agulia and Mandarim-de-Lacerda [2003]; Woods et al. [2003]; Kobayasi et al. [2010]), and are widely used for MS study (Frigolet et al. [2013]).

The dipeptidyl peptidase IV (DPP IV) is a serine-type proteinase (EC 3.4.14.5) which metabolizes peptide hormones, such as glucagon-like peptide-1 (GLP-1), an insulinotropic peptide hormone can stimulate glucose-dependent insulin secretions (Mentlein [2005]; Drucker [2006]; Idris and Donnelly [2007]). DPP IV inhibitors that control the glycermia by modulating the GLP-1 levels are currently developed for type 2 DM treatments (Smyth and Heron [2005]; Idris and Donnelly [2007]). DPP IV cleaves the Pro or Ala at the second position of the active *N*-terminal GLP-1(7–37) or GLP-1(7–36) amide which results in functional inactive GLP-1(9–37) or GLP-1(9–36) amide (Drucker [2006]; Idris and Donnelly [2007]). Therefore, researchers attempted to isolate potential DPP IV inhibitory peptides from protein hydrolysates, such as pepsin-pancreatin hydrolysates of sodium caseinate, skim milk powders and milk protein concentrates (Lacroix and Li-Chan [2012]), Umamizyme G hydrolysates of defatted rice bran (Hatanaka et al. [2012]), and pepsin-treated whey protein (Lacroix and Li-Chan [2013]).

Yams are recognized as herbal plants since the tuber dried slices are widely used as Chinese herbal medicines, and the fresh tuber has also been a staple food in West Africa, Southern Asia, and the Caribbean. The tubers of yam storage protein, dioscorin, account for about 90% of the extractable water-soluble proteins from different species as estimated by the immune staining method (Hou et al. [2000]). The yam dioscorin and/or its peptic hydrolysates have been reviewed for biological activities in vitro and/or in vivo (Lu et al. [2012]), among which the antihypertensive activities (Hsu et al. [2002]; Lin et al. [2006]; Liu et al. [2009a],[b]; Lin et al. [2014]) and antioxidant activities (Hou et al. [2001]; Liu et al. [2006]; Han et al. [2013], [2014a],[b],[c]) may involve in MS studies. Therefore, a HF diet which the fat composition provide 60% of total calories is used to induce obese rats and then yam dioscorin is intervened to observe the changes of body weights, blood pressures, and glucose tolerances. It is also to test the pepsin hydrolysates of yam dioscorin to evaluate DPP IV inhibitory activities.

## Methods

### Materials

DPP IV (lyophilized powder, ≥ 10 units/mg protein, D-7052), glucose, Gly-Pro *p*-nitroanilide, pepsin (3460 units/mg solid, P-6887), and sitagliptin phosphate were purchased from Sigma Chemical Co. (St. Louis, MO). The HF diet for obesity induction (fat composition provide 60% of total calories, D12942) was purchased from Research Diets, Inc. (NJ, USA). The standard mouse/rat chow (fat composition provide 12.137% of total calories, Prolab® RMH2500, 5P14 Diet; PMI Nutrition International, MO, USA).

### HF diets-induced obese rats for MS studies

Male 10-week-old Wistar rats (N = 24) were purchased from National Laboratory Animal Center (Taipei, Taiwan) and housed in wire-bottomed stainless steel cages in a temperature- and humidity-controlled room (at 22°C) with a 12-h light/dark cycle which had free access to the feeds and water. All animal experimental procedures were reviewed and approved by the Institutional Animal Care and Use Committee, Taipei Medical University (LAC-100-0038). After acclimation for one week, rats were randomly divided into three groups (N = 8 for each group), including a blank group for standard mouse/rat chow and two HF diet-induced obese groups (one control as HF group and one dioscorin-intervened group) for 70-days. In the dioscorin intervened group, yam dioscorin (50 mg/kg, dio50) was intervened daily concurrent HF diets (HF + dio50) from day-36 to day-70. The rat weights and feed intakes were recorded during experiments. The feed conversion is defined as a ratio of amounts of feed intakes (g) divided by rat weight gains (g) during experimental periods. Rats were sacrificed, organs (heart, lung, liver, kidney, and spleen) and adipose tissues (retroperitoneal fat, mesenteric fat, visceral fat, and testicle fat) were collected and weighted for comparisons.

### Changes of blood pressures in the dioscorin intervention of HF diet-induced obese rats

The systolic blood pressure (SBP) and diastolic blood pressure (DBP) of rats were measured at the end of each week after dioscorin intervention for 1-, 2-, 3-, and 4-weeks by using an indirect tail-cuff blood pressure meter (BP-98A, Softron Co. Ltd. Tokyo, Japan). Distilled water (0.5 ml) was administered to the rats in the normal diet group (the blank) and HF group (the control) instead of dioscorin solutions for comparisons.

### Impaired glucose tolerances in the dioscorin interventions of HF diet-induced obese rats by oral glucose tolerance tests

Impaired glucose tolerance of HF diet-induced obese rats were measured by oral glucose tolerance tests (OGTT) following the previous report (Ito et al. [2001]; Andrikopoulos et al. [2008]) with modifications. Rats of each group at the ends of the 10^th^-week were fasted for 16 hours and glucose was administered by oral gavage at 1 g/kg body weight. Blood (0.1 ml) was obtained from the tail vein of rats at 0, 5, 30, 60, 90, and 120 min after the glucose loads. Plasma glucose were determined by using RANDOX glucose kit (Randox Laboratories-US, Ltd. USA). The assay principle is based on the hydrogen peroxide generation catalyzed by glucose oxidase and further reacted with phenol and 4-aminophenazone to produce reddish violet quinoneimine dye with absorbance at 505 nm.

### DPP IV inhibitory activities of peptic hydrolysates of yam dioscorin

The procedure for peptic hydrolysates of yam dioscorin was following the previous reports (Hsu et al. [2002]; Liu et al. [2006]). The hydrolytic ratio (wt/wt) of yam dioscorin (g) to pepsin (g) was set at 5/1 in 0.1 M KCl buffer (pH 2.0) with stirring at 4°C for 3 days. After hydrolysis, 1.0 M Tris–HCl buffer (pH 8.3) was added to pH 7.5 to stop hydrolysis. The molecular cut-off centricon or centriprep device (YM-10, 10 kD; YM-3, 3 kD; Millipore Co., USA) was used to separate peptic hydrolysates of yam dioscorin into three portions as followings: higher than 10 kD (peptide > 10 kDa), between 3 to 10 kD (3 kD < peptide < 10 kD), and less than 3 kDa (peptide < 3kD). The fractions of between 3 to 10 kD (3 kD < peptide < 10 kD) and less than 3 kDa (peptide < 3kD) were further analyzed by reverse-phase C18 Spherical HPLC column (10 mm × 250 mm). The mobile phase was mixed in stepwise gradients with solvent A (deionized water containing 0.1% trifluoroacetic acid) and solvent B (100% acetonitrile containing 0.1% trifluoroacetic acid) as followings, 0 to 5 min, 100% to 79% solvent A and 0 to 21% solvent B; 21 min, 75% solvent A and 25% solvent B; 22 min to 27 min, 60% solvent A and 40% solvent B; 40 min, 0% solvent A and 100% solvent B. Flow rate was 3 ml/min. The detector was set at 220 nm. The latter two fractions were lyophilized for DPP IV inhibitory assays. The DPP IV inhibitory activity was assayed according the previous report (Lacroix and Li-Chan [2013]) with modifications. The DPP IV enzyme powder (≥10 units/mg protein) was re-dissolved in 1 ml of 100 mM Tris buffer (pH 8.0) as a stock enzyme solution and a 50-fold dilution before uses as a working enzyme solution. The substrate, Gly-Pro *p*-nitroanilide, was prepared as 4 mM stock solutions. The sitagliptin phosphate, the DPP IV inhibitor (Drucker et al. [2007]) as the positive control, was prepared as 1 μM stock solutions. Each 50 μl of working DPP IV enzyme solution and peptic dioscorin fractions was mixed at 37°C for 10 min, then 50 μl of substrate was added and adjusted to 200 μl by 100 mM Tris buffer (pH 8.0). The sitagliptin phosphate was used instead of tested dioscorin fraction for the control. The 100 mM Tris buffer (pH 8.0) was used instead of tested dioscorin fraction for the blank. The absorbance at 405 nm was measured at 60 min by using an ELISA reader (TECAN Sunrise microplate reader; Männedorf, Switzerland). The DPP IV inhibition (%) was calculated as (A405_blank_-A405_sample or control_/A405_blank_) × 100%.

### Statistical analyses

Data were expressed as mean ± SEM in the rat weight changes and impaired glucose tolerances by OGTT methods, others were expressed as mean ± SD. Multiple group comparisons under the same treated time were performed using one-way analysis of variance (ANOVA) followed by the *post hoc* Tukey’s test, and values that have not been indicated with the same alphabet were significantly different (*P* < 0.05). Statistical analysis was performed using the GraphPad Prism 5.0 software (San Diego, CA, USA).

## Results

### Effects of dioscorin interventions on body weights of HF diet-induced obese rats

**Figure 1:**
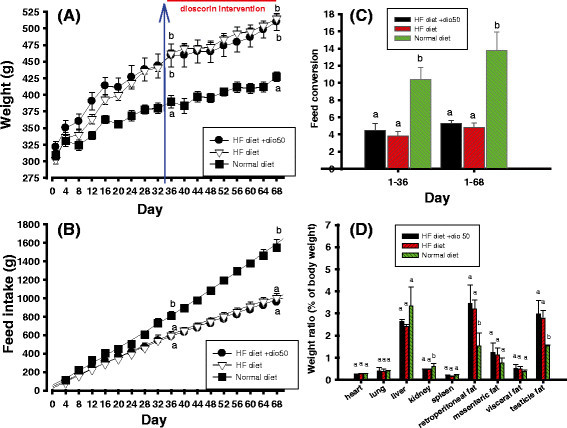
**Effects of dioscorin interventions (50 mg/kg) on (A) body weights, (B) feed intakes, (C) feed conversion (feed intakes/weight gains), and(D) weights of organ and fat tissues of high fat-diet induced obese rats.** Arrow indicates the dioscorin intervention from day 36 to day 70. Data were expressed as mean ± SEM in the rat weight changes and others were expressed as mean ± SD. Multiple group comparisons under the same treated time were performed using one-way analysis of variance followed by the *post hoc* Tukey’s test, and values that have not been indicated with the same alphabet were significantly different (*P* < 0.05). HF, a high-fat diet; (HF diet + dio50), yam dioscorin (50 mg/kg, dio50) was intervened daily concurrent HF diet.

At the day 36, the average weight of rats in the group of normal diet, HF diet, and (HF diet + dio50) was 389.65 ± 22.79 (g), 462.25 ± 11.06 (g), and 459.32 ± 38.94 (g), respectively. At the day 68, the average weight of rats in the group of normal diet, HF diet, and (HF diet + dio50) was 427.17 ± 18.20 (g), 514.90 ± 13.44 (g), and 509.76 ± 28.54 (g), respectively. Rats fed with the standard chow (the normal diet) showed lighter average body weights and significant difference (*P* < 0.05) compared to those fed with HF diets without or with dioscorin interventions during 70-day experiments (Figure 1A). While, the average weight of rats in two groups of HF-induced obese rats, namely HF diet group or with yam dioscorin interventions (HF diet + dio50) group showed no significant differences (*P* > 0.05) at the beginning (day 36) and the ends (day 68) of experiments (Figure 1A). It meant that yam dioscorin interventions at 50 mg/kg for 5 weeks showed no weight reduction activity against obese-induced rats at the present protocol designs. Figure 1B showed the feed intakes during experiments. The feed intakes in three rat groups were showed linearly increases, while, rats fed with the standard chow (the normal diet) showed higher feed intakes and significant difference (*P* < 0.05) compared to those fed with HF diets without or with dioscorin interventions during 70-day experiments (Figure 1B). Figure 1C showed the feed conversion rate of three rat groups during obesity-induced and the whole experimental stages which was calculated from feed intakes (data in the Figure 1B) divided by weight gained (data in the Figure 1A). Rats fed with the standard chow (the normal diet) showed higher feed conversion rate and significant difference (*P* < 0.05) compared to those fed with HF diets without or with dioscorin interventions during obesity-induced and the whole experimental stages (Figure 1C). It meant that more of standard chow (the normal diet) were needed compared to those of HF diet to gain the same weight. Figure 1D showed the organ weights of three rat groups. Generally, rats in the normal diet group showed significant difference (*P* < 0.05) and lower weights of adipose tissues (such as retroperitoneal fat and testicle fat) than those in the HF diet groups. Other organs except from kidney showed no significant difference among three groups (*P* > 0.05).

### Effects of dioscorin interventions on blood pressures of HF diet-induced obese rats

**Figure 2:**
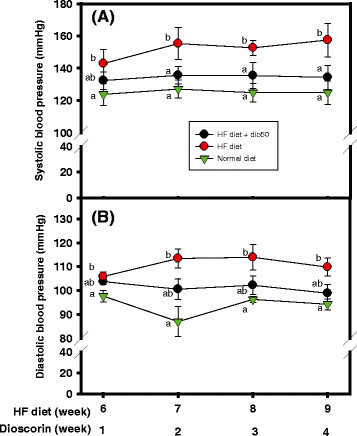
**Effects of dioscorin interventions (50 mg/kg) on (A) systolic blood pressure and (B) diastolic blood pressures of high fat-diet induced obese rats.** Blood pressure was measured at the end of each week after dioscorin intervention for 1-, 2-, 3-, and 4-weeks by using an indirect tail-cuff blood pressure meter (BP-98A, Softron Co. Ltd. Tokyo, Japan). Data were expressed as mean ± SD. Multiple group comparisons under the same treated time were performed using one-way analysis of variance followed by the *post hoc* Tukey’s test, and values that have not been indicated with the same alphabet were significantly different (*P* < 0.05). HF, a high-fat diet; (HF diet + dio50), yam dioscorin (50 mg/kg, dio50) was intervened daily concurrent HF diet.

Figure 2 showed the changes of SBP (Figure 2A) and DBP (Figure 2B) in three rat groups after obesity-induced stages (day 36 to day 70). From the results of Figure 2A, the rats in the normal diet group showed lower SBP and significant differences (*P* < 0.05) compared to those in the HF diet group at 6-, 7-, 8-, and 9-week. While, dioscorin intervention group (HF diet + dio50) showed lower SBP and significant differences (*P* < 0.05) compared to those in the HF diet group at 7-, 8-, and 9-week (corresponding to dioscorin intervention for 2 weeks, 3 weeks, and 4 weeks, respectively) and comparable to those in the normal diet group. From the results of Figure 2B, the rats in the normal diet group showed lower DBP and significant differences (*P* < 0.05) compared to those in the HF diet group at 6-, 7-, 8-, and 9-week. Dioscorin intervention group (HF diet + dio50) showed lowered SBP, however, did not exhibit significant differences (*P* > 0.05) compared to those in the HF diet group at 7-, 8-, and 9-week. These results showed that the dioscorin intervention could improve blood pressure in obese rats, especial for SBP.

### Effects of dioscorin interventions on impaired glucose tolerances of HF diet-induced obese rats

**Figure 3:**
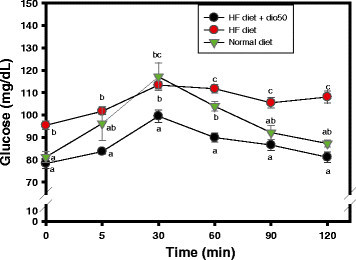
**Effects of dioscorin interventions (50 mg/kg) on impaired glucose intolerances of high fat-diet induced obese rats by oral glucose tolerance tests.** Data were expressed as mean ± SEM. Multiple group comparisons under the same treated time were performed using one-way analysis of variance followed by the *post hoc* Tukey’s test, and values that have not been indicated with the same alphabet were significantly different (*P* < 0.05). HF, a high-fat diet; (HF diet + dio50), yam dioscorin (50 mg/kg, dio50) was intervened daily concurrent HF diet.

After fasting overnight, the impaired glucose tolerance of rats in three groups were measured by OGTT (Figure 3). Plasma glucose concentrations (mg/dL) of the normal diet group in the 0, 5, 30, 60, 90, and 120 min were 81.19 ± 6.09, 96.22 ± 18.67, 117.13 ± 15.22, 103.90 ± 5.22, 92.14 ± 7.57, and 87.24 ± 3.77, respectively; plasma glucose concentrations (mg/dL) of HF diet group were 95.40 ± 5.66, 101.67 ± 4.07, 113.39 ± 5.04, 111.75 ± 5.51, 105.48 ± 7.02, and 108.05 ± 8.34, respectively; plasma glucose concentrations (mg/dL) of (HF diet + dio50) group were 78.31 ± 6.66, 83.66 ± 3.06, 99.50 ± 8.42, 89.92 ± 6.08, 86.59 ± 7.52, and 81.14 ± 7.21, respectively. At the beginning, rats in the normal diet group and (HF diet + dio50) group showed lower plasma glucose concentrations and significant differences (*P* < 0.05) compared to those in the HF diet group. Later, rats in the (HF diet + dio50) group kept the lowest plasma glucose concentrations and significant differences (*P* < 0.05) among three rat groups at each 5-min, 30-min, 60-min, 90-min, and 120-min time intervals and comparable to rats in the normal diet group at 90-min and 120-min time intervals. The plasma glucose concentrations of rats in the normal diet groups and (HF diet + dio50) group were then back to the baseline at 120 min, however, the plasma glucose concentrations of rats in the HF diet group were still kept at higher levels (108.05 ± 8.34 mg/dL). From the OGTT data, it meant that the dioscorin intervention could improve impaired glucose tolerances of HF diet-induced obese rats.

### DPP IV inhibitory activities of peptic hydrolysates of yam dioscorin

**Figure 4:**
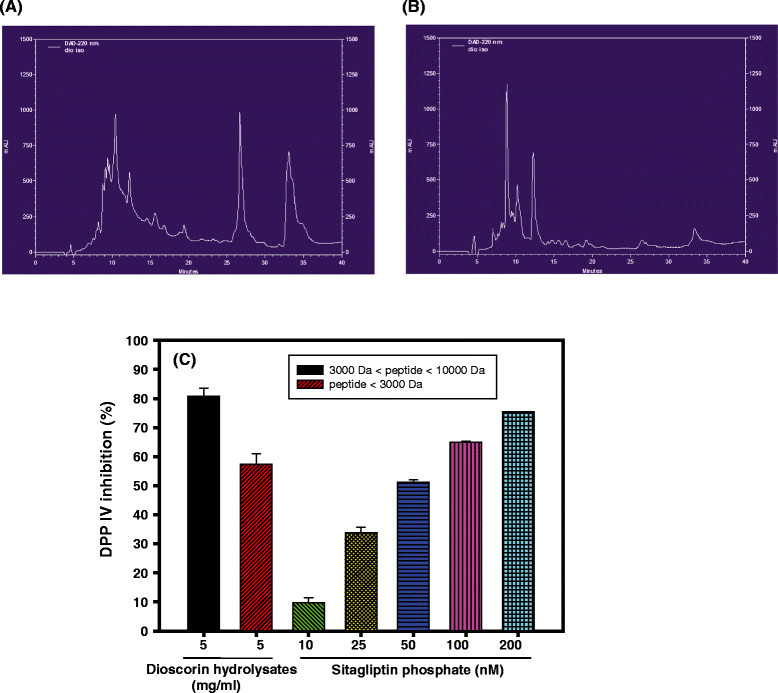
**DPP IV inhibitory activities of peptic hydrolysates of yam dioscorin. (A)** HPLC chromatograms of yam dioscorin peptic hydrolysates with molecular mass between 3 kD and 10 kD, **(B)** HPLC chromatograms of yam dioscorin peptic hydrolysates with molecular mass less than 3 kD, and **(C)** DPP IV inhibitory activities of two fractions (5 mg/ml) of dioscorin peptic hydrolysates from molecular cut-off membrane devices, and sitagliptin phosphate was acted as positive controls.

The reverse phase HPLC chromatograms of peptic hydrolysates of yam dioscorin with molecular mass between 3 kD and 10 kD (3 kD < peptide < 10 kD, Figure 4A) and less than 3 kD (peptide < 3kD, Figure 4B) were shown at Figure 4. These two hydrolytic fractions were used to analyze the DPP IV inhibitory activities compared to the positive control of sitagliptin phosphate (Figure 4C). The sitagliptin phosphate showed dose-dependent inhibitions against DPP IV. Under 5 mg/ml doses, the peptide fractions of 3 kD < peptide < 10 kD and peptide < 3kD, respectively, showed 80.82 ± 2.68 (%) and 54.42 ± 3.51 (%) DPP IV inhibitory activities which might be equivalent to sitagliptin phosphate of 250.86 nM and 61.79 nM, respectively.

## Discussion

The present animal experiments showed that the dioscorin interventions at dose of 50 mg/kg daily for five weeks could improve SBP and impaired glucose tolerances, but had no effects on reductions of body weight and fat tissues, of 10-week HF diet-induced obese rats which the improved metabolic risks were involved in MS criteria. The dioscorin and its peptic hydrolysates at doses of 40 mg/kg were showed to have antihypertensive activities against spontaneously hypertensive rats (SHR) (Lin et al. [2006]); the dioscorin at doses of 20 mg/kg or 80 mg/kg showed to lower oxidative stress of BALB/c mice induced by subcutaneous galactose injections (Han et al. [2014b]). The obesity may play the central role in MS which the renin-angiotensin system in dysfunctional adipocytes will initiate inflammation and dyslipidemia, increase blood pressure and decrease insulin sensitivity accompanied with abnormal blood glucose (Frigolet et al. [2013]). Therefore, anti-obesity or prevention against obesity may reduce directly the risks of MS. There were several reports concerning anti-obesity activity from natural resources in rodent models induced by HF diets, such as α-lipoic acid (Kim et al. [2004]), cryptotanshinone from *Salvia militorrhiza* (Kim et al. [2007]), rutin and *o*-coumaric acid (Hsu et al. [2009]), honokiol and magnolol (Kim et al. [2013]), pectin pentaoligosaccharide (Li et al. [2013]), high taurine and glycine contents of scallop protein (Tastesen et al. [2014]). The above-mentioned models for HF diet-induced obese studies were generally applied either by HF diet pretreatment for a period of time and then tested sample interventions or co-treatment of HF-diet and tested samples in the same time. The vasorelaxing peptides of Arg-Phe and Ile-His-Arg-Phe derived from rice glutelin protein showed to lower food intakes in rodent models (Kagebayashi et al. [2012]; Kontani et al. [2014]) which maybe have anti-obesity activity. At present, it is not sure that less amounts of dioscorin used with the similar MS preventive activities, or higher amounts of dioscorin used or co-treatments of HF-diet and dioscorin used with anti-obesity activities. Reagan-Shaw et al. ([2007]) suggested to use body surface area normalization for dose translation from animal to human studies. It was calculated that the human equivalent dose was 8.11 mg/kg of human body weight from dioscorin intervention of 50 mg/kg of rat body weight in the present experiment. An adult of 60 kg weigh might have to consume about 490 mg dioscorin/day to achieve benefits of improving MS and need further investigations.

The yam dioscorin and its peptic hydrolysates or synthesized peptides derived from dioscorin had been reported to lower blood pressures using SHR as animal models (Lin et al. [2006]; Liu et al. [2009a],[b]; Lin et al. [2014]). From the present results of Figure 2, dioscorin intervention at least for two weeks showed to lower SBP of HF diet-induced obese rats and comparable to the normal diet fed ones. The dysfunctional adipocytes in obesity will increase circulating renin-angiotensin systems, and the generated angiotensin II from angiotensin I by angiotensin converting enzyme (ACE) hydrolysis resulted in the higher blood pressures (Frigolet et al. [2013]). It was reported that obesity may elevate systemic oxidative stress from overloaded nutrients of HF diets and advanced glycation endproduct generations (Kahn et al. [2006]). Glycation is the non-enzymatic modification of proteins through the reduction of sugars and their metabolized intermediates, such as glyoxal or methylglyoxal, and leads to the irreversible formation of advanced glycation end products (Kikuchi et al. [2003]). The hypertensive rat might increase oxidative stress and methylglyoxal amounts in vascular smooth muscle cells (Wu and Juurlink [2002]). The peptic hydrolysates of dioscorin showed ACE inhibitory activities (Hsu et al. [2002]) and the synthesized peptides derived from pepsin hydrolysates *in silico* vasorelaxing activities (Lin et al. [2014]). The synthesized peptides derived from pepsin hydrolysates *in silico* exhibited antioxidant and antiglycation activities in vitro and in vivo (Han et al. [2013]; Han et al. [2014a],[c]). It was proposed that the dioscorin intervention showed to lower SBP of HF diet-induced obese rats might be from ACE inhibitory and vasorelaxing activities, and in part from antioxidant and/or antiglycation activities of active peptides after dioscorin ingestions and need further investigations.

From the results of Figure 3, it was found that the impaired glucose tolerances in HF diet-induced obese rats were improved after dioscorin interventions by OGTT methods. At the beginning and the end of OGTT test, the obese rats exhibited significantly higher glucose levels compared to rats in normal diet group and (HF + dio50) group. From the results of Figure 4, it was found that yam dioscorin peptic hydrolysates with molecular mass 3 kD < peptide < 10 kD and less than 3 kD at doses of 5 mg/ml showed DPP IV inhibitory activities in vitro which might be equivalent to effects of 250.86 nM and 61.79 nM of sitagliptin phosphate, respectively. Moreover, the undigested dioscorin was also used to evaluate DPP IV inhibitory activity, under 5 mg/ml concentration, which showed 40.7% DPP IV inhibition (data not show). The native dioscorin showed less anti-DPP IV activity compared to the 3 kD < peptide < 10 kD and less than 3 kD of dioscorin peptic hydrolysates. The results of DPP IV activity of the native and digested dioscorin is in agreement with the general observation that short-chain biologically active peptides can be released and absorbed in the small intestine after oral administration (Phelan et al. [2009]) which may correlate with the improved OGTT. The sitagliptin phosphate, DPP IV inhibitor approved by US FDA in 2006, can prolong GLP-1 biological activities to stimulate glucose-dependent insulin secretions and for type 2 DM treatment in vivo (Idris and Donnelly [2007]; Drucker et al. [2007]). The DPP IV inhibitor (valine-pyrrolidide) showed the improved glucose tolerance and insulin secretion in HF diet-fed C57BL/6 J mice (Ahrén et al. [2000]). The mice fed a HF diet (58% fat) together with DPP IV inhibitor (NVP DPP728) in the drinking water for 8 weeks showed the improved glucose tolerance and increased circulating levels of insulin and GLP-1 compared to HF diet only (Reimer et al. [2002]). It was suggested that the improved glucose tolerance in HF diet-induced obese rats after dioscorin interventions might be in part from DPP IV inhibitory activities of active peptides from dioscorin after being ingested and need further investigations.

## Conclusions

In conclusion, yam dioscorin interventions exhibit the improved MS activities in obese rats and peptic hydrolysates of yam dioscorin in vitro exhibit DPP IV inhibitory activities which the related mechanisms may need further investigations.

## Authors’ original submitted files for images

Below are the links to the authors’ original submitted files for images. Authors’ original file for figure 1 Authors’ original file for figure 2 Authors’ original file for figure 3 Authors’ original file for figure 4
