# Supplementary figures and images for: Effects of yam dioscorin interventions on improvements of the metabolic syndrome in high-fat diet-induced obese rats
Source: Bot Stud. 2015 Feb 25;56:4. doi: 10.1186/s40529-015-0084-8 (PMC5432891; doi:10.1186/s40529-015-0084-8)

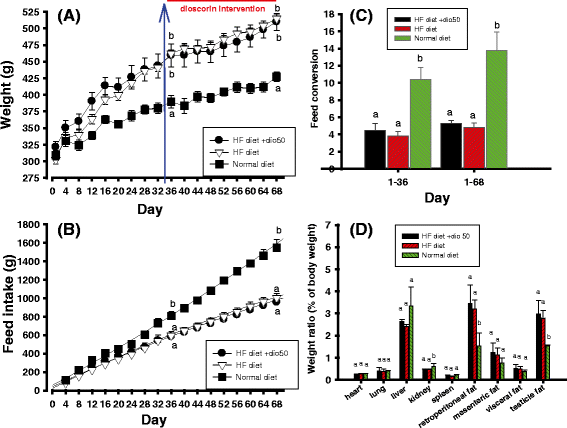

Supplement: Supplementary file 1 — Authors’ original file for figure 1 [file 40529_2015_84_MOESM1_ESM.gif]

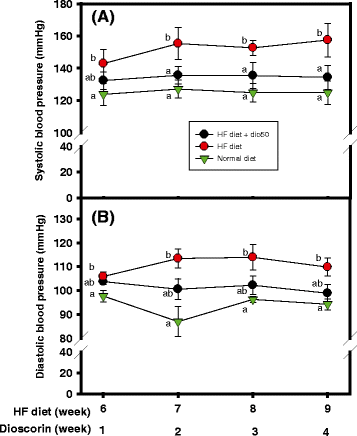

Supplement: Supplementary file 2 — Authors’ original file for figure 2 [file 40529_2015_84_MOESM2_ESM.gif]

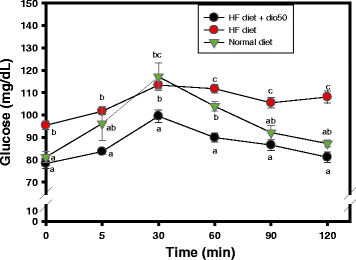

Supplement: Supplementary file 3 — Authors’ original file for figure 3 [file 40529_2015_84_MOESM3_ESM.gif]

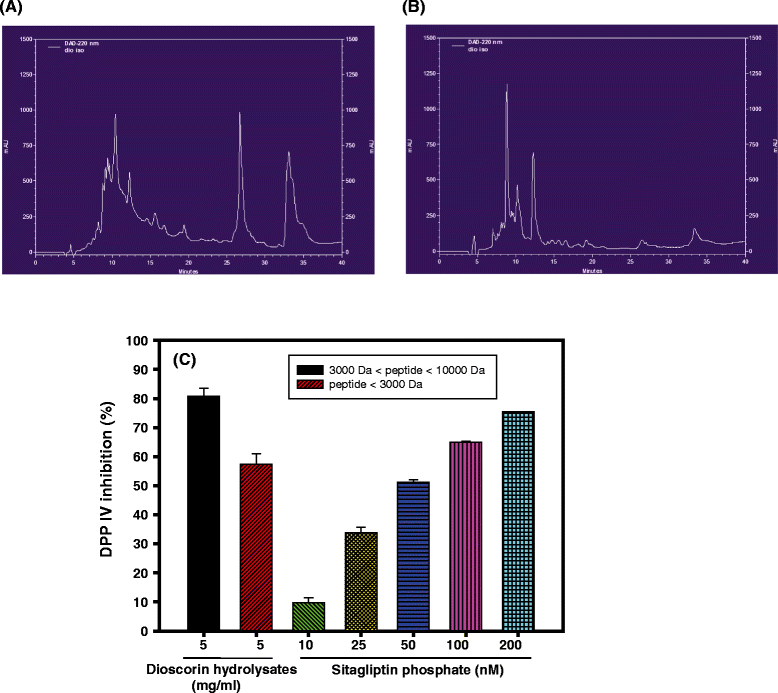

Supplement: Supplementary file 4 — Authors’ original file for figure 4 [file 40529_2015_84_MOESM4_ESM.gif]
